# The Metastatic Risk of Renal Cell Carcinoma by Primary Tumor Size and Subtype

**DOI:** 10.1016/j.euros.2023.04.015

**Published:** 2023-05-10

**Authors:** Steven M. Monda, Hansen T. Lui, Manolis A. Pratsinis, Thenappan Chandrasekar, Christopher P. Evans, Marc A. Dall'Era

**Affiliations:** a Department of Urologic Surgery, University of California Davis, Davis, CA, USA; b Department of Urology, Cantonal Hospital of St. Gallen, St. Gallen, Switzerland

**Keywords:** Renal cell carcinoma, Renal mass biopsy, Histology, Surveillance, Epidemiology and End Results, Active surveillance

## Abstract

**Background:**

Current data on the association between tumor size, subtype, and metastases, and thresholds for intervention, for renal cell carcinoma (RCC), are largely based on single-center nephrectomy registries that may under-represent those presenting with metastatic disease.

**Objective:**

We sought to assess tumor size and histologic subtype in relation to metastatic status at presentation for patients with RCC.

**Design, setting, and participants:**

Using Surveillance, Epidemiology and End Results cancer registry data, we identified patients with a diagnosis of RCC made between 2004 and 2019, and a known size of primary tumor. We used nodal and metastatic TNM staging to assess metastatic disease at presentation.

**Outcome measurements and statistical analysis:**

We report the proportion of metastatic disease across varying tumor sizes for clear cell (ccRCC), papillary (pRCC), and chromophobe (chRCC) RCC. We also examine sarcomatoid RCC and RCC with sarcomatoid features (sarcRCC). Logistic regression models were used to model the likelihood of metastatic disease for each histologic subtype.

**Results and limitations:**

Of 181 096 RCC patients included, 23 829 had metastatic disease. For any RCC, metastatic rates of 3.6%, 13.1%, 30.3%, and 45.1% were observed for tumors ≤4, 4–≤7, 7–≤10, and >10 cm, respectively. Metastatic rates of chRCC were low at even large sizes, 11.0% at >10 cm. In contrast, sarcRCC had high metastatic rates at all sizes, 27.1% at ≤4 cm. Metastatic rates for ccRCC and pRCC increased steadily above 3 cm. For any RCC and each evaluated subtype, tumor size was found to be associated with metastatic disease on logistic regression (*p* < 0.001).

**Conclusions:**

The likelihood of a renal mass being metastatic varies greatly with both its subtype and size. We report higher likelihoods of metastatic disease across tumor sizes compared with what has been reported previously. These results may help clinicians pick appropriate thresholds for intervention and candidates for active surveillance.

**Patient summary:**

We find that the metastatic probability of renal cell carcinoma varies greatly with subtype and increases with tumor size.

## Take Home Message

The likelihood of a renal mass being metastatic increases significantly with size but also varies widely with histologic subtype. Examining cancer registry data, we report a higher likelihood of metastatic renal cell carcinoma across all tumor sizes compared with what has been reported previously.

## 1 Introduction

With the increased use of cross-sectional imaging, incidental renal masses are being diagnosed at an increasing frequency, among older patients, and at smaller sizes [1], [2]. Many of these masses are indolent, and a growing body of evidence supports the long-term safety of active surveillance for appropriately selected renal mass patients [3], [4]. Active surveillance has been incorporated into major guidelines [5], [6]. Appropriate selection weighs oncologic risk and treatment options against patient comorbidities, surgical risk, and life expectancy.

Increasingly, biopsy has been used in select situations to supplement tumor size and growth kinetics in making treatment decisions [7], [8]. Yet, there is a scarcity of data comparing metastatic rates of renal cell carcinoma (RCC) for varying tumor sizes. To date, studies assessing the metastatic risk of RCC across tumor sizes and for different subtypes are based largely on single-center nephrectomy cohorts—cohorts limited in their representation of those presenting with metastatic disease [9], [10].

An updated analysis assessing the metastatic status of RCC across tumor size and for specific subtypes is needed to better counsel patients and select candidates for active surveillance. Our aim is to establish metastatic risk across tumor sizes for all RCC (any RCC) and the most common RCC subtypes using cancer registry data. We sought to establish the rates of metastatic disease at presentation for any RCC, clear cell RCC (ccRCC), papillary RCC (pRCC), chromophobe RCC (chRCC), as well as sarcomatoid RCC and RCC with sarcomatoid features (sarcRCC).

## 2 Patients and methods

Using Surveillance, Epidemiology and End Results (SEER), we identified patients older than 18 yr with a diagnosis of RCC made between 2004 and 2019, and a known size of the primary ranging from 0.5 to 20 cm. RCC patients were identified using International Classification of Diseases for Oncology 3 (ICD-O-3) histology coding. Histologic subtypes were identified using validated definitions within SEER, and only patients coded as RCC “not otherwise specified” were included in our any RCC reporting given the variable histology within this coding [11]. Coding for each histologic subtype can be found in Supplementary Table 1. Tumors with sarcomatoid histology or sarcomatoid features (sarcRCC) were also specifically assessed given the availability of these data and the uniquely poor prognosis of these tumors [12]. A patient with ccRCC could be included in both the ccRCC and the sarcRCC group if their ccRCC had sarcomatoid features. Nodal and metastatic status from TNM staging was used to define metastatic disease. Tumor size was defined as per the SEER convention from either the pathology report of the resected primary tumor or, if the primary tumor was not resected, the maximal diameter observed on radiographic imaging prior to therapy [13].

Cohort characteristics were summarized with medians and interquartile ranges (IQRs), or with incidences and percentages. Percentages of patients with metastatic disease were reported in 1-, 2-, and 3-cm increments for tumors up to 8, 14, and 20 cm, respectively, given the decreasing sample sizes of these larger tumors. Two-sided 95% confidence intervals (CIs) are reported for these percentages.

Logistic regression models were used for each subtype to model the likelihood of metastatic disease using tumor size and sex as independent variables. Sex has been established as a predictor of aggressive subtype and was thus included in our model [14]. The 95% CI of hazard ratios (HRs) and the significance of *p* < 0.001 were reported. All analyses were performed in R version 4.2.1.

## 3 Results

Cohort characteristics and demographics of our included 181 096 patients are reported in Table 1. Of the patients, 64% were male. A total of 23 829 patients (13%) had metastatic disease at presentation. The median age was 63 yr (IQR = 54–72). The median tumor size was 3.9 cm in patients with localized RCC and 8.1 cm in patients with metastatic RCC.

**Table 1:** Cohort characteristics

Variable	Localized (*n* = 157 267) Median (IQR) or *n* (%)	Nonlocalized (*n* = 23 829) Median (IQR) or *n* (%)
Age (yr)	63 (54–72)	65 (57–74)
Tumor size (cm)	3.9 (2.5–6.0)	8.1 (5.8–11.0)
Year of diagnosis		
Female sex	57 681 (37)	7590 (32)
Race		
White	128 450 (82)	19 819 (83)
Black or African American	17 536 (11)	2251 (9)
Asian or Pacific Islander	8376 (5)	1398 (6)
American Indian or Alaskan Native	1594 (1)	285 (1)
Hispanic	23 501 (15)	3695 (16)
Income ($)		
+75 000	46 384 ((29)	6486 (27)
60 000–75 000	60 787 (39)	9206 (39)
45 000–60 000	35 954 (23)	5837 (24)
<45 000	14 135 (9)	2298 (10)
Intervention for primary		
Ablation	9643 (6)	130 (1)
Nephrectomy (radical or total)	80 207 (51)	9738 (41)
Partial nephrectomy	50 994 (32)	450 (2)
None	15 893 (10)	13 385 (56)
Histology		
ccRCC	91 326 (58)	108 321 (45)
pRCC	20 101 (13)	1300 (5)
chRCC	9350 (6)	267 (1)
RCC (not otherwise specified)	36 490 (23)	11 431 (48)
sarcRCC a	2409 (2)	2403 (10)
Grade b (*n* = 123 508)		
1	13 753 (12)	331 (3)
2	56 883 (51)	2126 (18)
3	26 736 (24)	3658 (30)
4	4663 (4)	2390 (20)
Unknown	9391 (8)	3626 (30)

ccRCC = clear cell RCC; chRCC = chromophobe RCC; IQR = interquartile range; pRCC = papillary RCC; RCC = renal cell carcinoma; sarcRCC = sarcomatoid RCC and RCC with sarcomatoid features.

a Including primary sarcomatoid histology as well as any RCC with sarcomatoid features.

b Grade included only for pRCC and ccRCC.

The observed percentages of patients with metastatic disease at given tumor sizes are reported in Table 2. For any RCC, a metastatic rate of 3.6% was observed for tumors ≤4 cm, 13.1% for tumors 4–≤7 cm, 30.3% for tumors 7–≤10 cm, and 45.1% for tumors >10 cm.

**Table 2:** Proportion of patients with metastatic disease for a given size of primary tumor

Tumor size (cm)	Any RCC % of patients (95% CI)		ccRCC % of patients (95% CI)		pRCC % of patients (95% CI)		chRCC % of patients (95% CI)		sarcRCC % of patients (95% CI)	
0.5–<2	2.7%	(2.5–3.0%)	1.6%	(1.3–1.9%)	1.2%	(0.9–1.7%)	0.6%	(0.3–1.5%)	34.1%	(26.3–42.7%)
2–<3	2.5%	(2.3–2.7%)	1.5%	(1.3–1.7%)	1.3%	(1.0–1.7%)	0.8%	(0.4–1.4%)	22.8%	(16.8–30.2%)
3–<4	4.0%	(3.8–4.3%)	2.5%	(2.3–2.8%)	2.5%	(2.0–3.0%)	0.9%	(0.5–1.5%)	25.6%	(20.0–32.1%)
4–<5	7.1%	(6.8–7.4%)	4.8%	(4.5–5.2%)	4.0%	(3.4–4.9%)	1.1%	(0.7–1.9%)	32.0%	(27.0–37.4%)
5–<6	11.5%	(11.0–12.0%)	8.1%	(7.5–8.6%)	6.5%	(5.5–7.7%)	2.2%	(1.4–3.4%)	41.3%	(36.4–46.4%)
6–<7	16.7%	(16.1–17.3%)	12.6%	(11.9–13.4%)	9.4%	(7.8–11.1%)	2.4%	(1.4–3.8%)	45.0%	(40.4–49.7%)
7–<8	22.6%	(21.8–23.4%)	17.9%	(17.0–18.9%)	12.1%	(10.1–14.5%)	3.5%	(2.1–5.6%)	45.9%	(41.3–50.6%)
8–<10	30.0%	(29.2–30.7%)	25.4%	(24.5–26.3%)	16.7%	(14.6–19.0%)	4.9%	(3.5–6.7%)	53.5%	(50.1–56.8%)
10–<12	40.3%	(39.3–41.4%)	36.5%	(35.1–37.8%)	23.4%	(20.2–26.8%)	6.0%	(4.2–8.6%)	59.0%	(55.2–62.6%)
12–<14	46.1%	(44.7–47.5%)	42.7%	(40.9–44.6%)	28.4%	(24.1–33.1%)	12.3%	(9.0–16.4%)	64.9%	(60.4–69.2%)
14–<17	49.3%	(47.7–51.0%)	47.9%	(45.5–50.3%)	31.9%	(27.1–37.1%)	13.2%	(9.5–18.0%)	65.1%	(60.1–69.7%)
17–≤20	49.4%	(46.7–52.2%)	52.4%	(47.9–56.9%)	31.9%	(25.5–39.1%)	16.4%	(10.7–24.2%)	62.1%	(55.0–68.7%)

ccRCC = clear cell RCC; chRCC = chromophobe RCC; CI = confidence interval; pRCC = papillary RCC; RCC = renal cell carcinoma; sarcRCC = sarcomatoid RCC and RCC with sarcomatoid features.

The metastatic rates of ccRCC and pRCC varied greatly with tumor size, with ccRCC tending to have more metastatic disease at all tumor sizes than pRCC. For ccRCC, ≤4, 4–≤7, 7–≤10, and >10 cm tumors had metastatic rates of 2.1%, 8.8%, 24.6%, and 41.9%, respectively. Whereas for pRCC, ≤4, 4–≤7, 7–≤10, and >10 cm tumors had metastatic rates of 1.7%, 6.7%, 16.0%, and 28.6%, respectively.

For chRCC, metastatic rates were low even at large tumor sizes, with only 4.3% of patients with tumors 7–≤10 cm and 11.0% of patients with tumors >10 cm having metastatic disease. In contrast, sarcRCC had high metastatic rates at all sizes, with 27.1% of patients with tumors ≤4 cm and 41.4% with tumors 4–≤7 cm having metastatic disease.

**Fig. 1:**
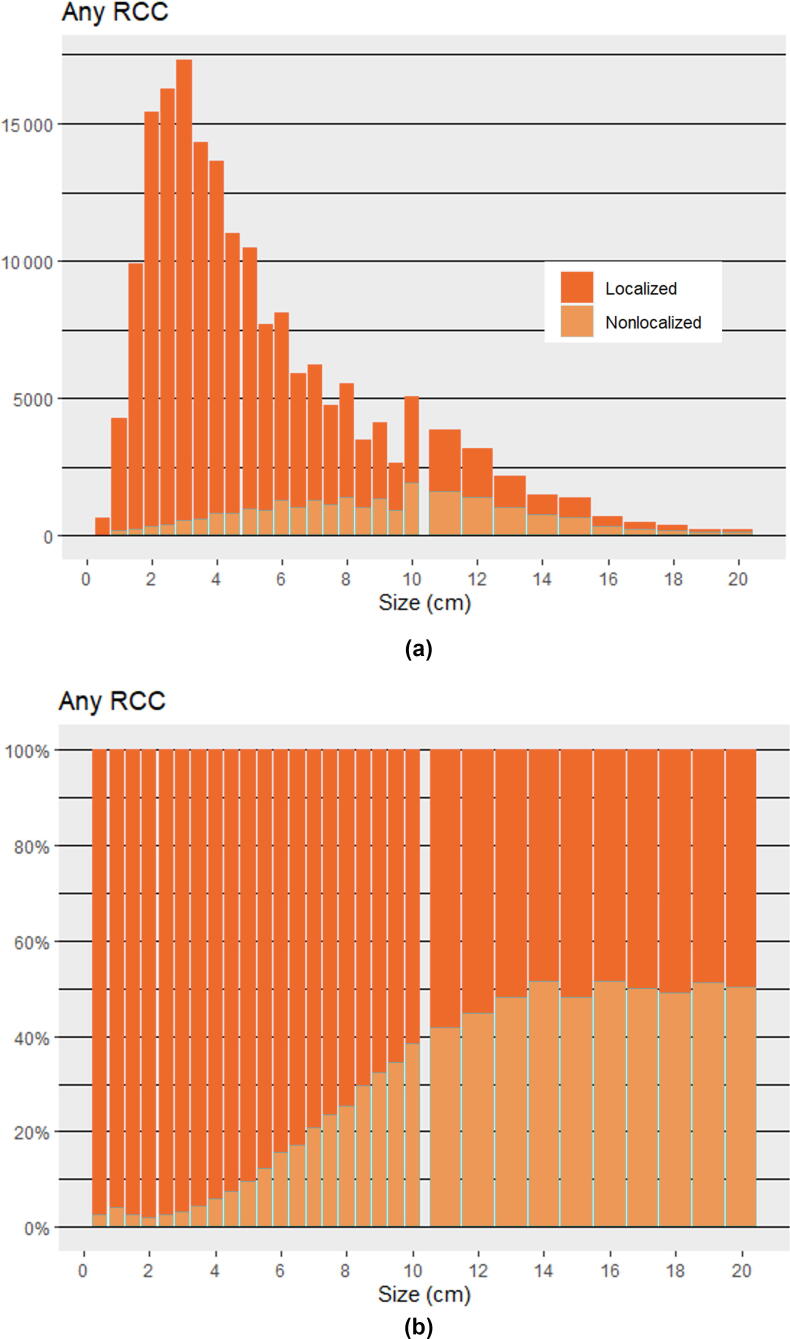
(A) Incidence and (B) proportion of localized and metastatic RCC at presentation for given tumor sizes. Listed for every 0.5 cm up to 10 cm and then every 1 cm up to 20 cm. RCC = renal cell carcinoma.

**Fig. 2:**
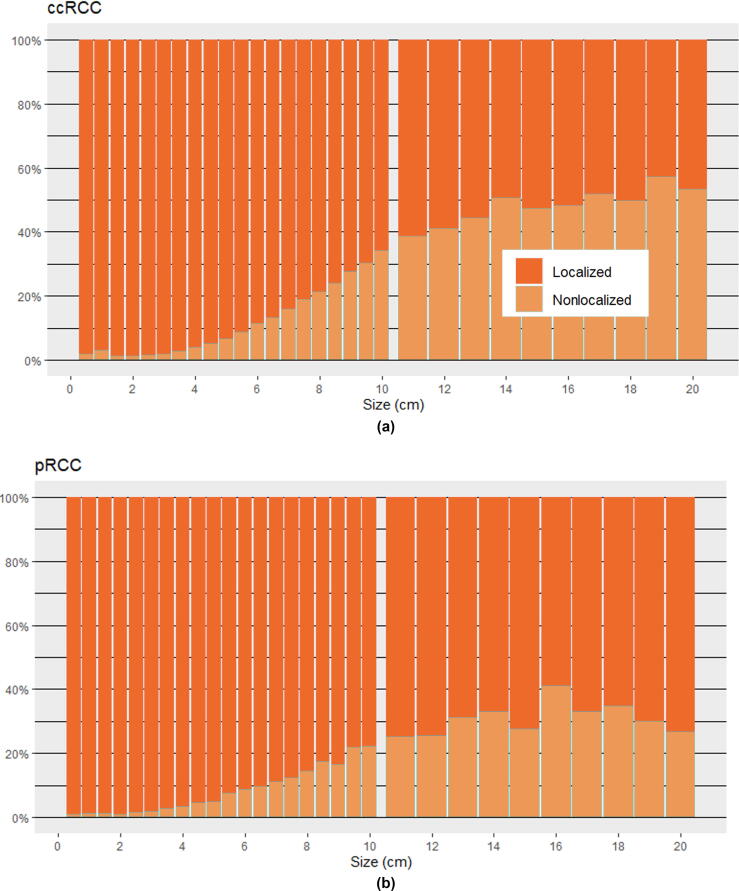

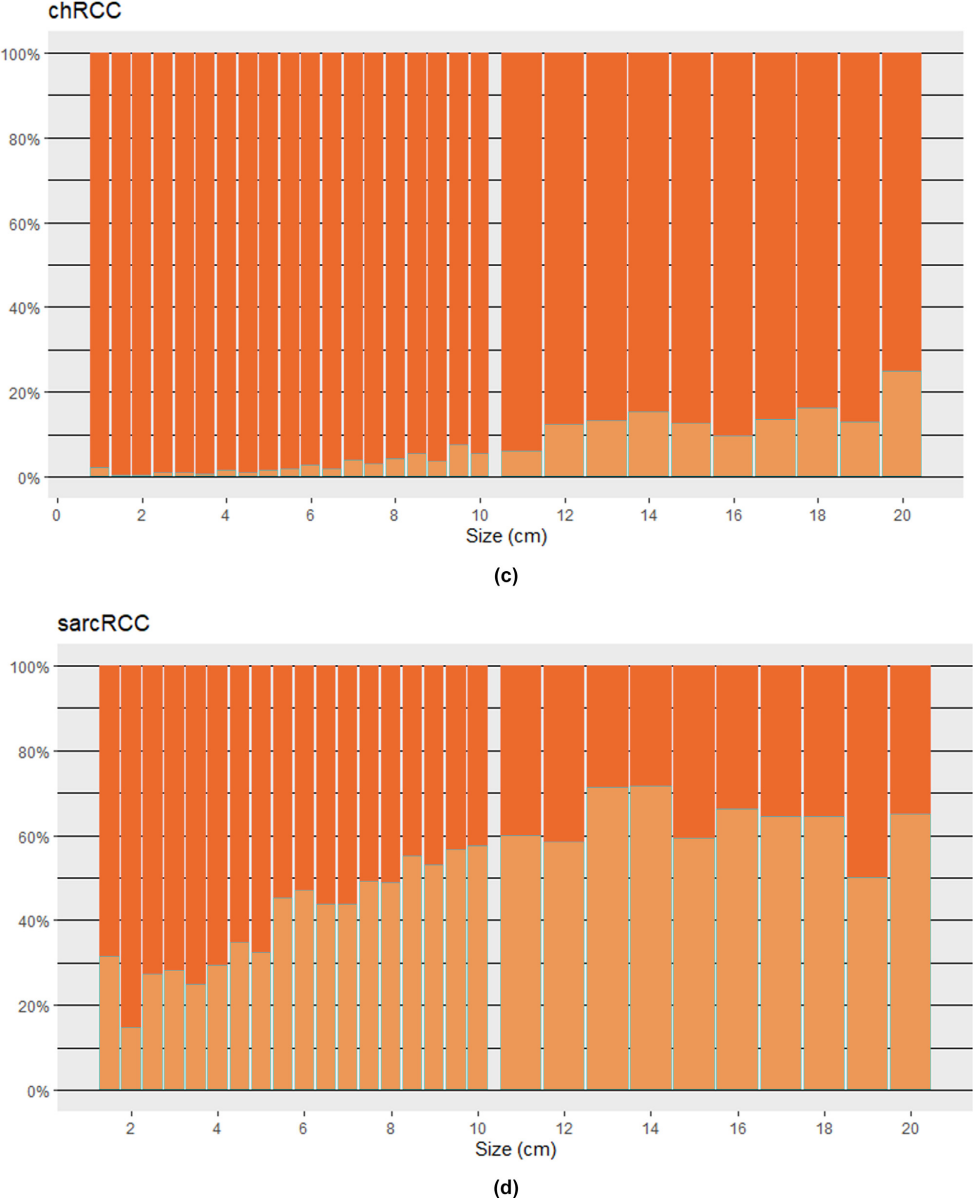
(A–D) Proportion of localized and metastatic RCC at presentation for specific subtypes and given tumor sizes. Listed for every 0.5 cm up to 10 cm and then every 1 cm up to 20 cm. ccRCC = clear cell RCC; chRCC = chromophobe RCC; pRCC = papillary RCC; RCC = renal cell carcinoma; sarcRCC = sarcomatoid RCC and RCC with sarcomatoid features.

Bar graphs with incidence and proportion of metastatic disease at varying tumor sizes are provided for any RCC in Figures 1A and 1B, and proportions for specific subtypes are provided in Figures 2A–D. Many patients in this study had small (≤4 cm) tumors that tended to be localized, and the number of patients in each centimeter group dropped rapidly with increasing size (Fig. 1A). Graphically, the metastatic risk for each histologic subtype tended to increase steadily for each centimeter above 3 cm. Although not part of our primary analysis, we report overall and RCC-specific survival curves for different tumor sizes in Supplementary Figures 1A and 1B to provide validation of our metastatic classification with a secondary clinical outcome.

The results of our logistic regression for any RCC and specific subtypes are given in Table 3. We report HRs attributed to tumor size and male sex. Tumor size was significantly associated with metastatic disease for all subtypes, with the highest HR for 1 cm change in tumor size observed for ccRCC (HR 1.38); sarcRCC, for which metastatic rates were high at even small tumor sizes, had the smallest HR associated with 1 cm change in tumor size (HR 1.12). Male sex was significantly associated with metastatic disease only for any RCC and ccRCC.

**Table 3:** Logistic regression models for the likelihood of metastatic disease

	Size (+1 cm) HR (95% CI)	Male sex HR (95% CI)
Any RCC	1.34 * (1.33–1.34)	1.13 * (1.10–1.17)
ccRCC	1.38 * (1.37–1.39)	1.24 * (1.18–1.30)
pRCC	1.29 * (1.27–1.30)	0.99 (0.85–1.13)
chRCC	1.24 * (1.21–1.27)	1.08 (0.84–1.39)
sarcRCC	1.12 * (1.11–1.14)	1.09 (0.97–1.24)

ccRCC = clear cell RCC; chRCC = chromophobe RCC; CI = confidence interval; HR = hazard ratio; pRCC = papillary RCC; RCC = renal cell carcinoma; sarcRCC = sarcomatoid RCC and RCC with sarcomatoid features.

* *p* < 0.001.

## 4 Discussion

In this study, we report the proportion of patients with metastatic disease at presentation among different RCC subtypes across varying primary tumor sizes. Our results are consistent with prior studies based on nephrectomy registries establishing the aggressive natures of specific subtypes and the indolent nature of others [9], [10], [15], [16]. We supplement this prior work with the use of SEER cancer registry data to include patients who may never have been considered for nephrectomy.

We find that chRCC has a low metastatic risk up to large tumor sizes, supporting observation in certain patients with oncocytic neoplasms. The risks of pRCC and ccRCC vary with size, with substantial risk differences at current T1a, T1b, T2a, and T2b size thresholds. The metastatic risk appears to increase steadily as tumors grow larger than 3 cm and starts to plateau at very large tumor sizes, roughly 12 cm. We observed that sarcRCC has a high metastatic risk even when very small, supporting the known poor prognosis associated with this disease [17].

The metastatic rates we observed in this study of the SEER registry are roughly two-fold that reported in prior nephrectomy registry studies [9], [10]. We suspect that this difference in results reflects a selection bias in nephrectomy registries, a surgical subset of patients less likely to have metastatic disease at presentation. Indeed, only 44% of the patients with metastatic disease in our cohort underwent initial intervention (ablation or surgery) for their primary tumor compared with 90% of patients with localized disease. Although our study included only RCC, other studies have included benign lesions in their analysis of renal masses, which reduces the likelihood of metastatic disease in those results. Nevertheless, for tumors 4–7 cm, 90% of which were malignant, Umbreit et al [9] reported only a 4% metastatic rate, far less than the 13% we observed for this size range for any RCC. Our inclusion of nodal disease in our definition of metastatic may also have increased the metastatic rates that we observed. However, only 11% of metastatic patients in our study had nodal disease without distant metastasis, suggesting that this inclusion did not increase our rates substantially.

Our logistic regression established higher odds ratios for metastatic disease for each centimeter change in tumor size for ccRCC and then pRCC, followed by chRCC and sarcRCC. In chRCC, where the tumors are mostly indolent, and in sarcRCC, where the tumors are very aggressive, the influence of a centimeter is apparently less pronounced. However, for ccRCC, a centimeter changes the metastatic rate considerably. We controlled for sex in our logistic regression given prior work suggesting the association of sex with aggressive disease [14]. However, we only saw a metastatic association with male sex in any RCC and ccRCC, and not in other subtypes, suggesting that ccRCC alone may be driving this association.

This work does not establish any causality; tumor size is associated with tumor biology, and more aggressive tumors tend to be larger and grow faster. Size alone is not responsible for worse prognosis.

Data on the aggressiveness of pRCC versus ccRCC vary but suggest better survival in patients with pRCC than in those with ccRCC in nephrectomy cohorts but worse survival in metastatic cohorts [18], [19]. We observe slightly lower rates of metastasis at given tumor sizes for pRCC than for ccRCC. This suggests, along with the prior literature, that pRCC may be less likely to metastasize at a given size but behaves worse once metastatic, perhaps due to less systemic therapy options.

This study is limited by the accuracy of its population registry data. Still, tumor size has independently been validated in SEER for RCC, as well as other cancers, with satisfactory results, and tumor size inaccuracies that were present in earlier versions of SEER have largely been corrected in more recent versions [20], [21]. Other important radiographic features of RCC such as venous invasion and lack of a clear capsule could not be accounted for in this study. RCC histology has been validated within SEER with high specificity and moderate sensitivity for ccRCC, pRCC, and chRCC. By excluding RCC (“not otherwise specified”) from the histologic subtype analysis, we sought to minimize misclassification [11].

As per the SEER and pathologic convention, tumor grade, for pRCC and ccRCC, cannot be established from biopsy of a metastatic site. For this reason, a large proportion of pRCC and ccRCC patients presenting with metastatic disease in our cohort, who did not have resection of the primary tumor, had unknown grades. Although we demonstrate grade proportion at each tumor size for the subset of pRCC and ccRCC of known grade in Supplementary Figures 2A and 2B, we did not include any statistics on grade because of the roughly 30% of metastatic pRCC and ccRCC tumors for which grade was not known. Given the known intratumor heterogeneity of RCC and the related shortcomings of biopsy specifically in characterizing tumor grade, we feel that the absence of a grade analysis does not represent a substantial weakness of our study [22], [23].

The outcome of synchronous metastasis at the time of diagnosis, as examined in this study, does not address the risk of micrometastases and long-term metachronous recurrence, both of which are clinically important and likely reduced with intervention at smaller tumor sizes. Still our study provides an updated analysis on the rates of metastatic RCC at presentation across tumor sizes, a clinically important outcome, which we hope will help guide decisions around intervention. Particularly, as biopsy becomes more integrated into clinical practice, knowing subtype-specific metastatic rates will allow us to provide more accurate counseling to our patients.

## 5 Conclusions

The metastatic risk of RCC varies significantly with tumor size and subtype. Our results suggest that the metastatic risk may be higher than reported previously for all tumor sizes. The risk of metastasis increases steadily with size in tumors larger than 3 cm for pRCC and ccRCC. The risk of presenting with metastatic disease for chRCC, even with a large primary tumor, is very low, whereas the risk is high for sarcRCC, even with a small primary tumor.

  ***Author contributions*:** Steven M. Monda had full access to all the data in the study and takes responsibility for the integrity of the data and the accuracy of the data analysis.

  *Study concept and design*: Monda, Dall’Era.

*Acquisition of data*: Monda.

*Analysis and interpretation of data*: Monda, Lui.

*Drafting of the manuscript*: Monda, Lui, Pratsinis.

*Critical revision of the manuscript for important intellectual content*: Dall’Era, Lui, Pratsinis, Chandrasekar.

*Statistical analysis*: Monda.

*Obtaining funding*: Pratsinis.

*Administrative, technical, or material support*: Monda, Dall’Era, Evans.

*Supervision*: Dall’Era, Evans.

*Other*: None.

  ***Financial disclosures:*** Steven M. Monda certifies that all conflicts of interest, including specific financial interests and relationships and affiliations relevant to the subject matter or materials discussed in the manuscript (eg, employment/affiliation, grants or funding, consultancies, honoraria, stock ownership or options, expert testimony, royalties, or patents filed, received, or pending), are the following: None.

  ***Funding/Support and role of the sponsor*:** Manolis Pratsinis acknowledges support from the Swiss Cancer Research Foundation (BIL KFS-5248-02-2021).

## Figures and Tables

**Supplementary Fig. 1a:**
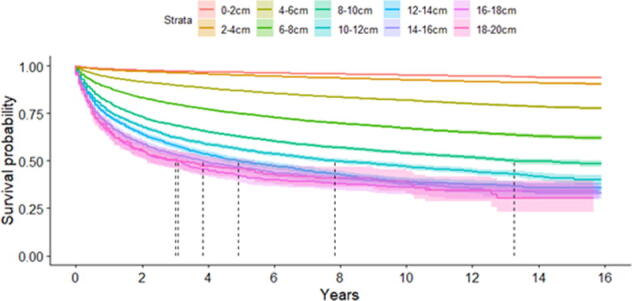
RCC-specific and overall survival curves for any RCC for a given size of primary tumor. Vertical dashed lines represent median survival where reached.

**Supplementary Fig. 1b:**
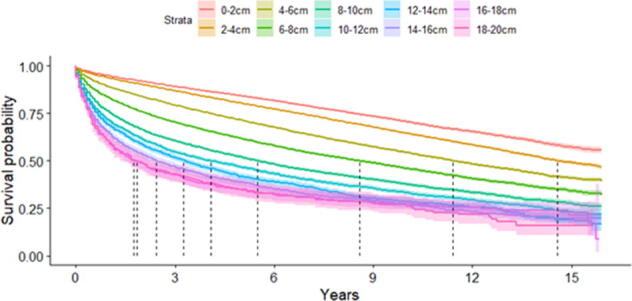
RCC-specific and overall survival curves for any RCC for a given size of primary tumor. Vertical dashed lines represent median survival where reached.

**Supplementary Fig. 2a:**
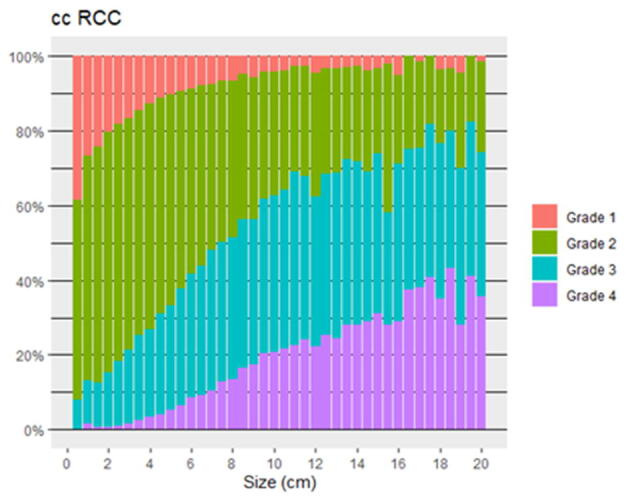
(A and B) Proportion of ccRCC and pRCC with Grade 1- 4 histology for a given tumor size.

**Supplementary Fig. 2b:**
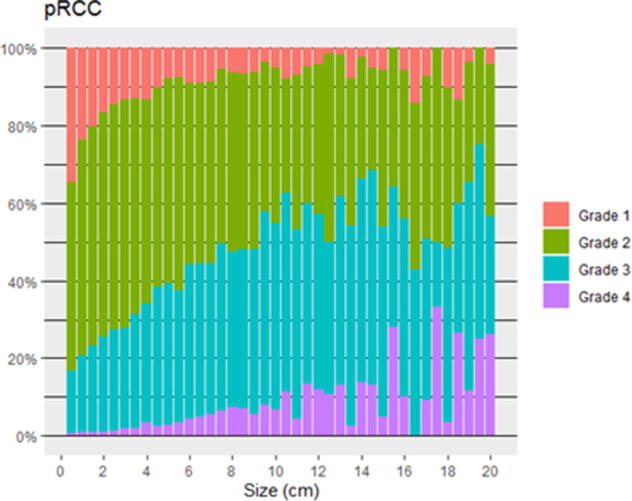
(A and B) Proportion of ccRCC and pRCC with Grade 1- 4 histology for a given tumor size.

## References

[1] Capitanio U., Montorsi F. (2016). Renal cancer. Lancet.

[2] Sharp E., Guduru A., May A.M., Lombardo L., Siddiqui S.A., Hamilton Z.A. (2022). The distribution of metastatic renal cell carcinoma by presenting tumor stage in the modern era. Clin Genitourin Cancer.

[3] Smaldone M.C., Kutikov A., Egleston B.L. (2012). Small renal masses progressing to metastases under active surveillance: a systematic review and pooled analysis. Cancer.

[4] McIntosh A.G., Ristau B.T., Ruth K. (2018). Active surveillance for localized renal masses: tumor growth, delayed intervention rates, and >5-yr clinical outcomes. Eur Urol.

[5] EAU. Guidelines. Presented at the EAU Annual Congress Amsterdam; 2022. https://uroweb.org/guidelines/renal-cell-carcinoma.

[6] National Comprehensive Cancer Network, Inc. NCCN clinical practice guidelines in oncology (NCCN Guidelines®) for kidney cancer. V.3.2023. 2022. https://www.nccn.org/guidelines/guidelines-detail?category=1&id=1440.

[7] Ozambela M., Wang Y., Leow J.J., Silverman S.G., Chung B.I., Chang S.L. (2020). Contemporary trends in percutaneous renal mass biopsy utilization in the United States. Urol Oncol.

[8] Kutikov A., Smaldone M.C., Uzzo R.G., Haifler M., Bratslavsky G., Leibovich B.C. (2016). Platinum opinion renal mass biopsy: always, sometimes, or never?. Eur Urol.

[9] Umbreit EC, Shimko MS, Childs MA, et al. Metastatic potential of a renal mass according to original tumour size at presentation. BJU Int 2012;109:190–4; discussion 194. 10.1111/j.1464-410X.2011.10184.x 21557795

[10] Thompson R.H., Hill J.R., Babayev Y. (2009). Risk of metastatic renal cell carcinoma according to tumor size. J Urol.

[11] Shuch B., Hofmann J.N., Merino M.J. (2014). Pathologic validation of renal cell carcinoma histology in the Surveillance, Epidemiology, and End Results program. Urol Oncol.

[12] Browning L., Colling R., Verrill C. (2021). WHO/ISUP grading of clear cell renal cell carcinoma and papillary renal cell carcinoma; validation of grading on the digital pathology platform and perspectives on reproducibility of grade. Diagn Pathol.

[13] SEER. General rules | SEER training. https://training.seer.cancer.gov/collaborative/system/tnm/t/size/rules.html.

[14] Bhindi B., Thompson R.H., Lohse C.M. (2018). The probability of aggressive versus indolent histology based on renal tumor size: implications for surveillance and treatment. Eur Urol.

[15] Moch H., Gasser T., Amin M.B., Torhorst J., Sauter G., Mihatsch M.J. (2000). Prognostic utility of the recently recommended histologic classification and revised TNM staging system of renal cell carcinoma: a Swiss experience with 588 tumors. Cancer.

[16] Almdalal T., Karlsson Rosenblad A., Hellström M. (2022). Predictive characteristics for disease recurrence and overall survival in non-metastatic clinical T1 renal cell carcinoma—results from the National Swedish Kidney Cancer Register. Scand J Urol.

[17] Sun M., Shariat S.F., Cheng C. (2011). Prognostic factors and predictive models in renal cell carcinoma: a contemporary review. Eur Urol.

[18] Leibovich B.C., Lohse C.M., Crispen P.L. (2010). Histological subtype is an independent predictor of outcome for patients with renal cell carcinoma. J Urol.

[19] Dudani S., De Velasco G., Wells J.C. (2021). Evaluation of clear cell, papillary, and chromophobe renal cell carcinoma metastasis sites and association with survival. JAMA Netw Open.

[20] Nguyen M.M., Gill I.S. (2010). Coded tumor size may be unreliable for small metastatic renal cancers in the Surveillance, Epidemiology, and End Results dataset. Urology.

[21] Ehrenkranz R., Lam C., Petkov V.I. (2018). Quality assessment of tumor size data collection for pancreatic and breast cancer in SEER. J Registry Manag.

[22] Patel H.D., Johnson M.H., Pierorazio P.M. (2016). Diagnostic accuracy and risks of biopsy in the diagnosis of a renal mass suspicious for localized renal cell carcinoma: systematic review of the literature. J Urol.

[23] Gerlinger M., Rowan A.J., Horswell S. (2012). Intratumor heterogeneity and branched evolution revealed by multiregion sequencing. N Engl J Med.

