# Supplementary material for: The Metastatic Risk of Renal Cell Carcinoma by Primary Tumor Size and Subtype
Source: Eur Urol Open Sci. 2023 May 10;52:137–44. doi: 10.1016/j.euros.2023.04.015 (PMC10240521; doi:10.1016/j.euros.2023.04.015)
Supplement: Supplementary Table 1 [file mmc1.docx]

**Supplementary Table 1. ICD-0-3 histology codes**

| **Histology** | **ICD-O-3 Morphology Codes** |
| --- | --- |
| ccRCC | 8310 |
| pRCC | 8260 |
| chRCC | 8270, 8317 |
| sarcRCC* | 8318 |
| RCC (not otherwise specified)** | 8312, 8316 |
| *SEER also codes presence of sarcomatoid features as a separate variable to ICD-O-3. Any tumor noted to have these features was included in both sarcRCC and within their overlying histology.  **Given variable histology within this group, RCC (not otherwise specified) was only included within our any RCC reporting. | |
